# Treating Cardiovascular Disease with Liver Genome Engineering

**DOI:** 10.1007/s11883-022-00986-z

**Published:** 2022-03-01

**Authors:** Ayrea Hurley, William R. Lagor

**Affiliations:** Department of Molecular Physiology and Biophysics, Baylor College of Medicine, Houston, TX 77030 USA

**Keywords:** Cardiovascular disease, Lipid disorders, Somatic genome editing, CRISPR/Cas9, Liver-directed repair

## Abstract

**Purpose of Review:**

This review examines recent progress in somatic genome editing for cardiovascular disease. We briefly highlight new gene editing approaches, delivery systems, and potential targets in the liver.

**Recent Findings:**

In recent years, new editing and delivery systems have been applied successfully in model organisms to modify genes within hepatocytes. Disruption of several genes has been shown to dramatically lower plasma cholesterol and triglyceride levels in mice as well as non-human primates. More precise modification of cardiovascular targets has also been achieved through homology-directed repair or base editing. Improved viral vectors and nanoparticle delivery systems are addressing important delivery challenges and helping to mitigate safety concerns.

**Summary:**

Liver-directed genome editing has the potential to cure both rare and common forms of cardiovascular disease. Exciting progress is already being made, including promising results from preclinical studies and the initiation of human gene therapy trials.

## Introduction

Cardiovascular disease (CVD) is the leading cause of death worldwide, and most often originates from underlying atherosclerotic vascular disease. The liver plays a critical role in the production and clearance of circulating lipoprotein particles which determine an individual’s susceptibility to atherosclerosis. Elevated levels of cholesterol and triglycerides are both causal in the disease process and can be influenced by the combined effects of common genetic variants interacting with diet and lifestyle, as well as rare genetic variants with large effect sizes. Recent advances in genome editing technology have made it possible to precisely modify genes within hepatocytes with high efficiency. Targeting important metabolic genes within the liver is a promising strategy to treat both rare and common cardiometabolic disease. In each case, the risks and benefits to the patient must be carefully weighed, given the potentially permanent nature of the therapy. Here we review recent advances in somatic genome editing in the liver, specifically highlighting opportunities for the treatment of cardiovascular diseases.

## Methods for Gene Editing

**Figure 1:**
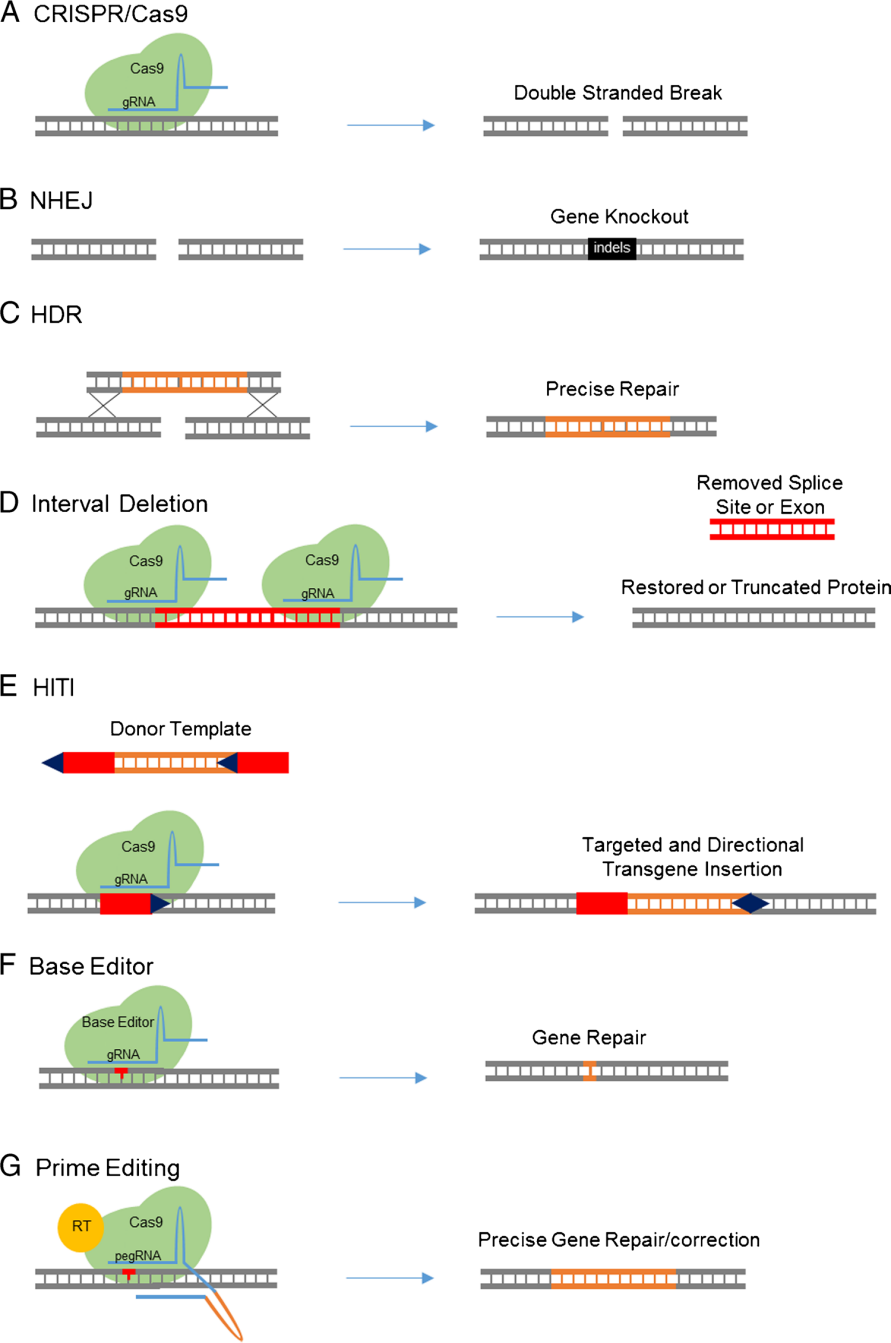
**A** CRISPR/Cas9 binds to DNA by a guide RNA and creates a double-strand break. Double-strand breaks can be repaired through **B** non-homologous end joining (NHEJ) creating indels or by **C** homology-directed repair (HDR) with a template for precise repair. **D** A splice site or exon can be removed by using two gRNAs to restore a normal or truncated version of the protein. **E** Homology-independent targeted integration (HITI) strategy uses gRNA sites in the donor template for directional transgene insertion. **F** Base editors can be used to change a single nucleotide without creating a double-strand break. **G** Prime editing uses a pegRNA which has the sequence template fused to the gRNA

The clustered regularly interspaced short palindromic repeat (CRISPR)–associated protein 9 (CRISPR/Cas9) system is an RNA-guided nuclease that has been adapted for gene editing [1–4]. CRISPR/Cas9 is guided to a target site in DNA through Watson-Crick base pairing with a complementary guide RNA (gRNA), where it creates a double-stranded break (DSB) (Figure 1A) [1–3]. DSB formation with CRISPR/Cas9 is sequence dependent since it requires engagement of the gRNA with the target site, and the downstream protospacer adjacent motif (PAM). With the requirement for most of the ~20 nucleotides of the gRNA to match the target, cutting of DNA with CRISPR/Cas9 can be highly specific with proper design and validation. Likewise, off-target cutting events can occur, but these are not random and involve high sequence similarity to the gRNA, typically with only 1–3 mismatches to the target site. The DSBs generated by CRISPR cutting can be repaired by two major DNA repair pathways known either as non-homologous end joining (NHEJ) (Figure 1B) or homology-directed repair (HDR) (Figure 1C).

The NHEJ pathway is an error-prone repair pathway active in all cells that most often results in small insertions and deletions (indels), although larger deletions and translocations can also occur at a lower frequency. Indels in coding sequences of exons can be used to shift the reading frame, resulting in a premature termination codon and nonsense-mediated decay of the mRNA — knocking out a gene. The NHEJ repair pathway can also be used to excise large intervening sequences of DNA by using two gRNAs, a strategy that can be useful for removal of regulatory elements, cryptic splice sites, or mutant exons (Figure 1D).

HDR is a more precise repair pathway that uses a donor template flanked by homologous sequences to insert new genetic material such as a single nucleotide variant, short sequence, exon, or even an entire gene. The efficiency of HDR is dramatically improved by using CRISPR/Cas9 to create a DSB near the insertion site [5–7]. Given the broad range of templates, HDR gene editing would be an ideal therapeutic approach as it could correct any defect in a given gene regardless of the many different underlying mutations in patients. A key limitation of HDR is that it only occurs in dividing cells [8], making it feasible for germline editing in model organisms, but impractical for adult liver where less than 1% of hepatocytes are actively dividing at a given time. Another limitation of HDR is that not every allele will be precisely repaired, and even in dividing cells, CRISPR/Cas9 cutting will also produce unintended indels through NHEJ repair.

Homology-independent targeted integration (HITI) is an alternative method to replace or insert large sequences of DNA with CRISPR/Cas9 cutting that bypasses the need for cell division with HDR [9, 10•]. In this approach, one or more DSBs are created with CRISPR/Cas9 at the intended insertion site. A donor template is supplied that is also cut on both ends with the same gRNA. The gRNA target sites in the donor template are oriented in an opposite direction relative to the insertion site in the genome. In this manner, backwards integrations will then restore the required gRNA cut site and result in their excision from the genome, thereby favoring integration in the correct orientation (Figure 1E). HITI is typically at least 10-fold more efficient than HDR in quiescent tissues, since the NHEJ repair pathway is active in both dividing and non-dividing cells. Disadvantages are that HITI is not always directional, and unintended scars can be left at each CRISPR cut site, including both small indels and larger deletions.

Base editors are synthetic proteins engineered to edit single nucleotides more precisely, without the requirement for a donor template or generation of DSBs. Base editors consist of Cas9 proteins fused to a deaminase that allows for the guided targeting and nicking of DNA for base replacement [11] (Figure 1F). The first two forms of base editing are the cytosine base editor (CBE) which converts C to T and conversely G to A [12], and the adenine base editor (ABE) which converts A to G and conversely T to C [11]. Base editors bind to DNA with a gRNA, but only nick one strand, greatly reducing the formation of indels [11–13]. Base editors have a limited window for editing within the gRNA binding site, so guides need to be carefully selected with this in mind. Bystander editing occurs when there is more than one of the target nucleotides within the editing window, which can be a challenging problem to overcome. Additionally, off-target effects can occur randomly with low frequency in both DNA and RNA since the activity of the deaminating enzyme is not entirely dependent upon Cas9/gRNA binding. Base editors can be used to correct many single nucleotide variants, which represent the majority of disease-causing mutations. Limitations of this approach are that not every base change is possible at this time, undesirable bystander edits can occur, and the gRNA must be carefully tailored for each specific mutation.

Prime editing is a very recent technology reported by Anzalone et al. in which short regions of sequences can be changed to any base using template-based reverse transcription from a modified gRNA scaffold [14••]. It is also possible to make precise insertions and deletions at least 80bp in size with the prime editing enzyme [14••]. The prime editor is a Cas9 nickase fused to a reverse transcriptase (RT). The prime editing guide RNA (pegRNA) consists of a primer binding site (PBS), RT template, the target DNA (sgRNA), and scaffold (Figure 1G). Briefly, the sgRNA binds to the target DNA, the nickase cuts the opposite strand, and the PBS attaches to the free end. The fused RT starts transcription of the RT template creating an edit on a 3′ flap. Through equilibrium, the 3′ flap rotates into the DNA creating the edited DNA with a 5′ unedited flap that can be cleaved. While prime editing allows for more flexible editing, there are many points to optimize. In addition, the first generation of prime editors use the large SpCas9 nickase [14••], which presents major challenges for viral delivery. One study shows that optimization is still needed for the smaller orthologs of Cas9, *Staphylococcus aureus* Cas9 (SaCas9) prime editing [15•]. Prime editing holds tremendous promise for editing and correction, but further work is needed to realize its full potential.

## Delivery Vehicles

Delivery efficiency is a critical factor in the success or failure of somatic genome editing approaches. The liver has evolved to serve as a filter for diet-derived nutrients, pathogens, toxins, and xenobiotics making it particularly amenable to the delivery of drugs, viral vectors, and nanoparticles. In addition, the porous nature of the fenestrated endothelium makes this organ a logical first target tissue. Here we will briefly outline two key modes of delivery to the liver for gene editing therapeutics.

Adeno-associated virus (AAV) is a non-enveloped single-stranded DNA virus that requires the presence of a helper virus for replication. Recombinant AAV can be produced that include only the transgene cassette flanked by the inverted terminal repeats (ITRs) on either side. The final product contains no viral genes and can deliver virtually any cargo within the packaging capacity of ~4.8kb [6, 16•, 17, 18, 19••, 20•, 21•, 22, 23••]. AAV vectors can be packaged with numerous naturally occurring or engineered capsids, almost all of which have a high tropism for the liver. Once inside the nucleus, recombinant AAV genomes are converted from single-stranded DNA to circular double-stranded episomes that provide stable expression from months to years. AAV has been used extensively for delivery of CRISPR/Cas9 in animal models. Due to the limited packaging capacity of AAV, *Staphylococcus aureus* Cas9 (SaCas9) [5] is often used. The advantages of AAV for delivery of genome editing machinery are the high efficiency for liver, a favorable safety profile, and a viable regulatory path for human gene therapy. Disadvantages of AAV include prolonged expression of the Cas9 nuclease and gRNA, pre-existing immunity to the AAV capsid which is frequent in humans, and theoretical cancer risks associated with rare but random integration events. Lastly, it has been increasingly appreciated that AAV vectors have a propensity to integrate at DSBs generated by genome editing nucleases [8, 22, 24–26].

Lipid nanoparticles (LNPs) are non-viral particles that are most often used for delivery of mRNA. LNPs are a promising delivery system due to the transient expression of Cas9 protein, which may last only a couple of days [27•, 28•, 29•, 30, 31•, 32•, 33, 34•, 35•]. The upper packaging limit is still unknown and there have not been significant immune responses detected. This is a major advantage, as it opens the door for delivery of much larger editing enzymes including base editors and prime editors, which are not possible with single AAV vectors. LNPs have also been used in combination with AAV [6, 27•] adding flexibility and the possibility or repeated dosing. Some other nanoparticles that have been used for gene editing in vivo are gold nanoclusters [36•], LipoMSN [37•], and nanoclew [38•]. Ribonucleoprotein complexes (RNPs) can also be used to deliver Cas9 protein along with a gRNA [28•, 29•, 31•, 32•, 37•, 38•], although additional moieties such as amphiphilic peptides or lipids are generally necessary for cellular uptake and endosomal escape.

## Targets for Disruption or Deletion

Since the NHEJ repair pathway is active in all cells, the most straightforward targets are genes that can be disrupted or inactivated. Below we highlight several such targets where genetic disruption could be used for therapeutic benefit.

Proprotein convertase subtilisin/kexin type 9 (PCSK9) is a secreted enzyme that binds to the low-density lipoprotein receptor (LDLR) and promotes its degradation. Gain-of-function mutations in the *PCSK9* gene result in familial hypercholesterolemia (FH), a disease with dramatically elevated low-density lipoprotein (LDL) cholesterol levels, due to an inability of the LDLR to recycle back to the cell surface. *PCSK9* is an excellent candidate for gene editing because inhibition of PCSK9 leaves LDLR free to internalize more LDL particles, dramatically lowering plasma cholesterol. Currently available inhibitors of PCSK9 are monoclonal antibodies (mAb) [39] and siRNAs [40]. Several mAbs have been shown to be highly effective and safe at reducing cholesterol levels; however, injections need to be repeatedly administered and this can be cost-prohibitive [39]. siRNA treatment has also been shown to be effective and safe at lowering cholesterol levels and injections are only needed twice a year [40]. Many studies have demonstrated that the in vivo disruption of the *Pcsk9* gene effectively lowers plasma cholesterol (Table 1), making it a favorite target for testing new gene editing nucleases and delivery systems.

**Table 1:** Studies of PCSK9 editing in animal models

**Species**	**Delivery vehicle**	**Editing method**	**% PCSK9 editing**	**% cholesterol reduction**	**References**
Mouse	AAV	CRISPR	30–50%	30–50%	[5, 16•, 17, 19••]
Mouse	AAV LNP	Base editor	>60%	>30% >40%	[34•]
Mouse	LNP	ZFN	>30%	Not reported	[27•]
Mouse	LNP	CRISPR	5–60%	Not reported	[29•, 31•, 35•]
Mouse	LNP	CRISPR	>80%	>30%	[30]
Mouse	Gold nanoclusters	CRISPR	>50%	30%	[36•]
Mouse	RNP	CRISPR	>20%	>30%	[37•]
Mouse	Nanoclew	CRISPR	>40%	>40%	[38•]
Mouse	Adenovirus	Base editor	5–20%	>20%	[41, 42•]
Mouse	Adenovirus	Base editor	>50%	Not reported	[43•]
Mouse	Adenovirus	CRISPR	>50%	>30%	[44]
Humanized mouse	AAV	CRISPR	>40%	No change	[18]
Humanized mouse	Adenovirus	Base editor	>5%	No change	[42•]
NHP	AAV	Meganuclease	10–50%	10–40%	[20•, 23••, 26]
NHP	LNP	Base editor	20–60%	10–60%	[28•, 34•]

Angiopoietin-like 3 (ANGPTL3) is a protein that inhibits lipoprotein lipase (LPL), the major enzyme responsible for clearance of triglycerides from the circulation. Loss-of-function mutations in *ANGPTL3* in humans are associated with decreased cholesterol, LDL, triglycerides, and reduced risk of CVD [45], providing strong genetic rationale for inhibition or disruption. Evinacumab is a mAb targeting ANGPTL3 that was recently approved for homozygous FH (HoFH) after clinical trial NCT03399786 showed a 49% reduction of cholesterol in HoFH patients [46•]. Other treatment options targeting ANGPTL3 include antisense oligonucleotides (ASOs) with >30% reduction of triglycerides (NCT04516291 and NCT02709850) [47•, 48], and an siRNA being tested on healthy volunteers (NCT03747224). So far, results show that the siRNA is well tolerated and shows a reduction in ANGPTL3 in a dose-responsive manner [49]. Somatic editing of *ANGPTL3* with CRISPR/Cas9 in mice showed therapeutic effects 100 days after a single injection and no toxicity in the liver [32•]. A base editor has also been used to edit *ANGPTL3* and reduce triglycerides in wild type (31%) and HoFH mice (56%) [12] making *ANGPTL3* a promising target for somatic gene editing.

Apolipoprotein C-III (ApoC3) inhibits LPL and is a negative regulator of triglyceride metabolism. Loss-of-function mutations in *APOC3* in humans result in lower triglycerides and reduced CVD risk [50, 51]. An siRNA targeting ApoC3 is being tested on healthy volunteers (NCT03783377) with results showing reduction of ApoC3 in a dose-responsive manner with only mild adverse events [49]. An ongoing trial (NCT03385239) using an ASO for ApoC3 in patients with CVD has shown improved lipid profiles [52]. Thus far, somatic disruption of *Apoc3* has only been studied in a mouse model in combination with knockdown of *Angptl3* and *Pcsk9* [37•]. Hamsters mimic important features of human lipoprotein metabolism and may be a more clinically relevant small animal model. CRISPR/Cas9 has been used to make an *Apoc3* knockout (KO) hamster model which showed reduced triglycerides and protection from atherosclerosis [53•], supporting *APOC3* as a promising target.

Lipoprotein(a) [Lp(a)] is an LDL-like particle with a protein called apolipoprotein(a) covalently attached to apolipoprotein B (ApoB)-100 through a disulfide linkage. Although usually only a minor subset of ApoB lipoproteins in the circulation, Lp(a) particles are particularly atherogenic through mechanisms that are not fully understood. The levels of Lp(a) are genetically determined by common haplotypes of the *LPA* gene encoding apolipoprotein(a), where individuals with many kringle repeats have slower rates of Lp(a) production by the liver, and consequently lower plasma levels and reduced CVD risk. In certain individuals, excessively high Lp(a) levels lead to accelerated coronary artery disease risk, effectively similar to monogenic disorders such as FH. While there are promising results in clinical trials (NCT04023552, NCT02160899, NCT02414594, and NCT03070782) for ASOs targeting the *LPA* gene [52, 54, 55•], no approved therapy currently exists. A recent clinical trial (NCT03626662) using an siRNA targeting Lp(a) is underway. Early results show sustained knockdown of Lp(a) after 113 days [56]. No known phenotype is associated with low or null Lp(a) levels [57] making this an excellent candidate for disruption, since lifelong correction could be achieved with a single dose.

## Targets for Precise Repair or Replacement

Many genes will require replacement or precise repair in the liver. Here we briefly highlight several potential therapeutic targets.

The LDLR binds to ApoB-lipoprotein particles and mediates their uptake by the liver through clathrin-mediated endocytosis. Mutations in the *LDLR* gene can cause FH and accelerated atherosclerotic disease [24]. While heterozygous FH can be well managed with statins and PCSK9 inhibitors, better treatment options are needed for compound heterozygous FH and HoFH. Over 1000 different LDLR mutations have been reported to cause FH, which are distributed throughout the entire gene and promoter regions [58]. Prime editing and base editing strategies may be viable, but the gRNA would have to be modified for every mutation, presenting major regulatory and manufacturing obstacles. Therefore, HoFH is an excellent candidate for editing approaches that involve gene replacement where a common transgene is inserted to correct the disease in many patients. One strategy involves replacement of larger exons in the gene through HDR in neonatal mice. Zhao et al. achieved impressive reductions in plasma cholesterol and atherosclerosis in mice treated with AAV-CRISPR vectors to deliver exon 4 [21•].

The rate-limiting enzyme in intravascular triglyceride hydrolysis is LPL [59]. Loss-of-function mutations in *LPL* cause increased triglycerides, CVD risk, chylomicronemia, and pancreatitis [59]. A gain-of-function mutation in the *LPL* gene forms a truncated protein [59] which was used to treat LPL deficiency in the first AAV gene therapy to receive regulatory approval in Europe [60]. Efficacy was suboptimal, likely owing to the targeted local delivery to the quadriceps. Nonetheless, LPL replacement could be used to lower triglycerides and protect these patients from life-threatening pancreatitis. Likewise, glycosylphosphatidylinositol-anchored high-density lipoprotein-binding protein 1 (GPIHBP1) is a binding partner for LPL, where loss-of-function mutations cause a similarly severe disease [59]. GPIHBR1 KO [61•] or mutant [62] mouse models have been generated using CRISPR to further study this protein. Targeted transgene insertion for *LPL* or *GPIHBPI* would likely need to occur in skeletal muscle rather than liver, to prevent unwanted hepatic fat accumulation.

Apolipoprotein C2 (ApoC2) is a secreted apolipoprotein that activates LPL. Mutations in this gene lead to hyperlipoproteinemia type IB, which is characterized by severe elevations in chylomicrons and plasma triglycerides even during fasting. The chronic hypertriglyceridemia in this disease is often accompanied by diabetes and life-threatening pancreatitis. Triglyceride clearance by LPL can be temporarily improved through plasma exchange or injection of peptidomimetics of ApoC2- which were first identified by Kinnunen et al. [63]. Interestingly, it has also been recently shown that both ANGPTL3 and ApoC3 inhibitors were effective at reducing triglycerides in a homozygous ApoC2 patient [64•]. Nonetheless, lifelong correction through gene replacement or gene editing would certainly be worthwhile. Recently reported hamster models [65•, 66•] will be useful in these efforts. Since ApoC2 is a liver-expressed secreted protein, it is likely that even modest degrees of gene editing or replacement in this organ could correct the disease.

## Recent Clinical Progress

The first liver-directed genome editing trial (NCT02695160) was initiated by Sangamo Therapeutics in 2016 to treat hemophilia B, a rare X-linked bleeding disorder. The trial involves AAV delivery of zinc finger nucleases (ZFN) that target the 3′ end of the highly expressed albumin gene for insertion of a secreted factor IX transgene. Since this time, Sangamo has also initiated two other clinical trials using the same approach for mucopolysaccharidosis types I (NCT02702115) and II (NCT03041324). There are many human proteins that have zinc finger domains, so it has been hypothesized that the engineered ZFN are less likely to provoke an immune response than bacterially derived nucleases such as Cas9. In addition, AAV was a logical choice for a delivery vehicle, given its safety profile and early success in other liver gene therapy trials. Despite the importance of these trials for the field, interim results suggest only modest efficacy [67]. Factors may include immune responses to the AAV capsid and difficulty in precisely modifying enough albumin alleles through HDR.

In vivo application of CRISPR/Cas9 for human therapeutics is already in progress in a trial by Editas to treat Leber congenital amaurosis (NCT03872479). The gene editing therapy uses an AAV vector to deliver SaCas9 to the retina. Two gRNAs are used to remove a pathogenic splice site mutation from intron 26 of the *CEP290* gene, restoring normal mRNA splicing from exon 26 to exon 27. Interim results appear promising. This trial is the first example of direct delivery of CRISPR/Cas9 to a diseased tissue in humans and will pave the way for others. Of particular interest will be information on the efficacy and durability of the therapy, and potential immune responses to the AAV capsid as well as SaCas9, which will be expressed indefinitely by the targeted cells.

Intellia initiated the first CRISPR/Cas9 liver-directed gene editing trial in November of 2019 to treat transthyretin amyloidosis (ATTR) (NCT04601051). The therapy uses LNP delivery of chemically modified mRNA encoding SpCas9 as well as gRNA targeting transthyretin (TTR). Indels in the TTR gene prevent production of the toxic misfolded protein by the liver [68••]. At 4 weeks post-injection, patients receiving the higher dose had an 87% reduction of TTR, no off-target editing, and only mild adverse effects [68••]. These incredibly promising results come on the heels of the success of the LNP-based Moderna and Pfizer vaccines for COVID-19 and show the tremendous potential of this technology to treat and prevent human diseases.

Verve Therapeutics is developing a liver-directed therapy for FH through disruption of *PCSK9*, using a base editor to avoid the undesirable on-target effects induced by DSB as well as LNPs to transiently deliver the base editor as mRNA. Thus far, they have tested ABE in macaques using a gRNA with an identical target in humans [28•]. This study showed sustained knockdown of PCSK9, decreased LDL cholesterol levels, and very little off-target effects 8 months post-injection. Acuitas Therapeutics were also successful in knockdown of PCSK9 in macaques after a single LNP dose of ABE [34•]. While the first application of this technology will almost certainly be for heterozygous FH, it could conceivably be applied far more broadly to more “garden variety” hyperlipidemias to lower the risk of death from CVD.

## Challenges and Unmet Needs

There has been an explosion of progress in the gene editing field over the past couple years which has culminated in multiple clinical trials. Along the way, our understanding of gene editing systems and the risks associated with these approaches continues to evolve.

Targeted disruption of a specific point mutation is a straightforward concept, and such therapies are already advancing into the clinic (i.e., TTR). If delivery challenges are solved, then one could imagine a regulatory path that affords flexibility in gRNA delivery, whereby each patient could be treated with the same nanoparticle system, differing only in the gRNA sequence. While this is conceptually appealing, each gRNA has a different cutting efficiency and risks of undesirable on-target and off-target modifications to consider. For diseases that require repair, a simplified strategy for repair would be advantageous.

In cases where there are many different mutations (i.e., LDLR), it would likely be preferable to perform targeted insertions of entire transgenes, to make the therapy generalizable to many patients. One of the main challenges is the low occurrence of HDR. Greater rates of integration can be achieved with HITI; however, this method generates a complicated mix of different editing events at the on-target site [9]. In either case, achieving highly efficient integration of the transgene across the entire liver is difficult. Recent work on methods to promote the selective expansion of correctly targeted cells may be able to solve this problem [43•, 69, 70•].

Achieving efficient delivery in a tissue and cell type-specific manner remains an ongoing challenge. Innovative approaches including rational design, capsid shuffling, peptide insertion, and biopanning are being combined with next-generation sequencing to generate AAV vectors with enhanced properties. Likewise, equally exciting advances are occurring for nanoparticle delivery, both with lipid and non-lipid based systems. For example, most lipid nanoparticles use a mixture of phosphatidylcholine, free cholesterol, a pegylated glycerolipid, and a cationic lipid [27•, 28•, 29•, 30, 31•, 32•, 33, 34•, 35•]. There are almost infinite possibilities for improvement in this design space, which include incorporation of novel lipids and peptides, different ratios of lipid constituents, and even the use of targeting moieties. Future work will yield a powerful toolkit for safe, efficient, and transient genome editing in a broad range of tissues beyond the liver.

A major unanswered question involves the risk of exposure to the bacterially derived Cas9 nuclease. Many people have pre-existing immunity to the two most commonly used Cas9 orthologs, SaCas9 and SpCas9. This pre-existing immunity includes both neutralizing antibodies, as well as memory T-cells. There is discordant data on the frequencies of pre-existing immunity [71••, 72•, 73••, 74], which is due to many factors, including variability in the subjects studied and the sensitivity and specificity of the assays. Based on clinical experience with AAV gene therapy, we have learned that even modest immune responses to the vector often determine the success or failure of a therapy. This is also likely to be the case with Cas9 therapeutics for the liver. The lessons learned from the ongoing trials, as well as careful work in model organisms, is critical to understand how the immune system will interface with this new class of therapeutics.

An important safety concern remains the risk of off-target editing. Over the past few years, there have been great advances in the prediction and identification of off-target events. For the CRISPR/Cas9 system, off-target events are believed to be dependent on gRNA binding. For base editing, there does appear to be a greater risk of randomly distributed mutations that do not depend on the sequence of the gRNA, but which are also more difficult to survey [75•]. Aside from off-target mutagenesis with the editing enzymes themselves, there is also a risk of off-target integration of vector sequences into the genome, even with primarily non-integrating viruses like AAV. Assessing the risks of off-target cutting and insertional mutagenesis is an extremely complex endeavor and must be tailored for each system and disease application. Importantly, unintentional germline editing or modification should be avoided at all costs.

## Conclusion

Many recent advances have been made in the field of gene editing for cardiovascular diseases. There are multiple attractive targets for liver-directed genome editing that could dramatically lower circulating lipid levels and reduce CVD risk. Editing enzymes and methods continue to undergo refinement, greatly improving the spectrum of mutations that can be corrected. Corresponding improvements in delivery systems, particularly viral vectors and nanoparticles, will enable translation to patients. While there is still much to learn, the future for this new class of therapeutics is bright.
